# RETRACTED: Erira et al. Differential Regulation of the EGFR/PI3K/AKT/PTEN Pathway between Low- and High-Grade Gliomas. *Brain Sci.* 2021, *11*, 1655

**DOI:** 10.3390/brainsci15060660

**Published:** 2025-06-19

**Authors:** Alveiro Erira, Fernando Velandia, José Penagos, Camilo Zubieta, Gonzalo Arboleda

**Affiliations:** 1 Facultad de Odontología, Universidad Cooperativa de Colombia, Bogotá 111311, Colombia; 2 Facultad de Medicina, Universidad del Rosario, Bogotá 111221, Colombia; fernando.velandia@urosario.edu.co; 3 Neuro Oncología, Instituto Nacional de Cancerología, Bogotá 111321, Colombia; pjpenagos@hotmail.com (J.P.); camilozubieta@hotmail.com (C.Z.); 4 Facultad de Medicina, Universidad Nacional de Colombia, Bogotá 111112, Colombia

The Journal retracts the article “Differential Regulation of the EGFR/PI3K/AKT/PTEN Pathway between Low- and High-Grade Gliomas” [1], cited above.

Following publication, concerns were brought to the attention of the publisher regarding the overlap of images presented in Figure 3.

Adhering to our standard procedure, an investigation was conducted that confirmed indications of inappropriate editing and duplication contained within the Western blots presented in Figure 3 [1] and Figure 3C [2], produced by a different authorship group.

While the authors cooperated during the investigation, they were unable to satisfactorily explain the overlap nor provide replacement images that meet the required quality standards for original images in order to warrant a correction as per the journal’s original image requirements policy (https://www.mdpi.com/journal/brainsci/instructions#oriimages (accessed on 1 April 2025)). As a result, the Editorial Board and Editor-in-Chief have lost confidence in the reliability of the findings and have decided to retract this paper as per MDPI retraction policy (https://www.mdpi.com/ethics#_bookmark30 (accessed on 1 April 2025)).

This retraction was approved by the Editor-in-Chief of the journal *Brain Sciences*.

The authors express their disagreement with the decision to retract this article. The authors wish to indicate that the similarities in parts of the Western Blots could be attributed to the fact that the research conducted by Erira et al. (2021) and Wang G et al. (2018) utilized the same standard laboratory technique under similar conditions. This work was previously presented, evaluated, approved, and published at a scientific event in 2013 [3].
